# RIOK1 is associated with non-small cell lung cancer clinical characters and contributes to cancer progression

**DOI:** 10.7150/jca.64668

**Published:** 2022-01-31

**Authors:** Rong Wang, Wen-Shu Chai, Dian-Zhu Pan, Li-Na Shan, Xuan Shi, Yu-Hai He, Shuang Pan

**Affiliations:** 1 Department of Respiratory, The First Affiliated Hospital Of Jinzhou Medical University, Jinzhou, Liaoning, 121001, China.; 2 Department of Gastroenterology, The First Affiliated Hospital Of Jinzhou Medical University, Jinzhou, Liaoning, 121001, China.; 3 School of Basic Medical Sciences, Jinzhou Medical University, Jinzhou, Liaoning, 121001, China.; 4 Department of Physiology, School of Basic Medical Sciences, Jinzhou Medical University, Jinzhou, Liaoning, 121001, China.

**Keywords:** RIOK1, non-small cell lung cancer, proliferation, invasion, chemical sensitivity

## Abstract

Lung cancer is one of the most common malignant tumors and is currently the leading cause of cancer-related deaths worldwide. Although the treatment strategy has been significantly improved, the prognosis of lung cancer patients is still quite poor. RIOK1 has been reported to be highly expressed in non-small cell lung cancer (NSCLC), however, its clinical significance and biological function are still largely unknown in lung cancer. Using western blot and immunohistochemistry, we showed that RIOK1 was highly expressed in NSCLC tissues and correlated with advanced stage and poor prognosis. Furthermore, knockdown of RIOK1 could inhibit proliferation, migration, and invasion in NSCLC cells and tumorigenesis *in vivo* through AKT, Cyclin B1, MMP2, and EMT pathway. Furthermore, cell viability and apoptosis assays demonstrated that RIOK1 maintained NSCLC cell survival and reduced apoptosis rate when cells were treated with cisplatin. Western blot analysis demonstrated that RIOK1 depletion caused up-regulated protein expression of cleaved PARP and Caspase-3 in NSCLC cells. These findings revealed a novel function of RIOK1 in non-small cell lung cancer progression and suggest that RIOK1 might become a promising diagnostic and therapeutic target for this disease.

## Introduction

Lung cancer is one of the most common malignant tumors and is currently the leading cause of cancer-related deaths worldwide 1, 2. Non-small cell lung cancer (NSCLC) accounts for about 85% of the total incidence of lung cancer 3. Proliferation and invasion are the most important factors leading to patient death in the malignant process of tumors 4. Therefore, identifying the key proteins that govern the malignant process of tumors and understanding the regulatory mechanisms play an important role in improving cancer patient survival.

RIO (right open reading frame) kinase is a highly conserved atypical serine/threonine-protein kinase family 5, 6. RIOK1 and RIOK2 are two important members of the RIO kinase family, named after S. cerevisiae Rio1p and Rio2p, respectively 5. RIOK3 is the third member of the RIO kinase family and has a high degree of similarity to RIOK1, which exists in multicellular eukaryotes 7. Unlike typical protein kinases, they exhibit weak kinase activity because of their lacking conserved activation loop motifs and substrate-binding domain 5, 8. RIOKs have been shown to have a pivotal role in promoting the maturation of small ribosomal subunits (SSU) 9-11, regulating cell cycle progression and cell division 12. In recent years, a growing number of researches focus on the function of RIOKs in tumorigenesis 13-18. RIOK2 siRNAs significantly inhibit the glial cell migration and invasion by down-regulating the expression of MMPs (MMP2 and MMP9) and mesenchymal markers (N-cadherin, β-catenin, Twist1, fibronectin, ZEB-1) 19. Rio Kinase 3 (RIOK3) changes the cytoskeleton structure and promotes the migration and invasion of pancreatic ductal cells by activating the small G protein Rac 20. RIOK3 can also promote hypoxia-driven tumor metastasis by maintaining the actin cytoskeleton organization required for migration and invasion 21. The high RIOK3 level in glioma promotes the proliferation, migration, and invasion of glioma cells through the AKT/mTOR pathway 22. In particular, the role of RIOK1 in tumors has been the most widely studied. RIOK1 is upregulated in 8% of primary cancer cells and has mutations in tumors suggesting it is a potential biomarker and anti-cancer drug objective 18, 23. The RIOK1 has been reported to engage in a functional relationship with constitutively active RAS to regulate the tumor cell malignancy 14. Over-expression of RIOK1 was further found in HR-negative breast cancer and correlated with advanced stage and poorer breast cancer patient prognosis. Overexpression of RIOK1 was shown to promote breast cancer cells proliferation and invasion by regulating the PI3K/AKT and MAPK/ERK signaling pathways in HR-negative breast cancer cells 17. In addition, RIOK1 has been reported to be highly expressed in NSCLC 23.

Although RIOK1 has been reported to be highly expressed in NSCLC, its clinical significance and biological function are still largely unknown. In our research, we show that RIOK1 is highly expressed in NSCLC and positively correlated with the malignant progression of lung cancer, and the high expression of RIOK1 indicates a poor prognosis. Furthermore, we also analyzed the effects of RIOK1 on the proliferation, migration, invasion, and chemical sensitivity of NSCLC cells and explored its mechanism of action to clarify its role in NSCLC.

## Materials and methods

### Patients and Tissue Samples

A total of 112 NSCLC tissue specimens selected after surgery from patients diagnosed with NSCLC at the First Affiliated Hospital of Jinzhou Medical University. Patients did not receive radiotherapy or chemotherapy before surgery. This study was approved by The First Affiliated Hospital of Jinzhou Medical University and the written informed consent was obtained from all the patients. All specimens were fixed with 4% neutral formaldehyde, embedded in paraffin, and stained with tissue sections to confirm the pathological diagnosis. It is according to the WHO classification guidelines to determine the clinicopathological stage of lung cancer. The expression of RIOK1 in pan carcinoma and associated normal tissues was performed by SangerBox (http://sangerbox.com/Index), a comprehensive tool for bioinformatics analysis based on R.

### Cell Culture and Transfection

HBE, H460, H661 and H1299 were purchased from the cell bank of Shanghai Chinese Academy of Sciences. A549 cell was a generous gift from Xueshan Qiu. Cells were maintained in RPMI 1640 medium (Gibco) supplemented with 10% FBS (fetal bovine serum), and were cultured at 37 °C in a humidified incubator at 5% CO_2_. RIOK1-RNAi-lentivirus was purchased from GeneChem Company (Shanghai, China). The shRNA RIOK1 sequence#1 was 5'-GTCATGAGTTTCATCGGTAAA-3'; the shRNA RIOK1 sequence#2 was 5'- GGCAAATAGAATGAGAACCAT-3'; and the shRNA control sequence was 5'- TTCTCCGAACGTGTCACGT-3'.

### Immunohistochemistry

Immunohistochemical (IHC) was performed on 4 μm paraffin sections, using an UltraSensitive^TM^ SP (Mouse/Rabbit) IHC Kit (Maixin Technology Co., Ltd, China). The primary antibody was rabbit polyclonal anti-RIOK1 (1:150, Abcam, Cambridge, UK). We evaluated the IHC staining results of lung cancer and adjacent tissues according to the established evaluation criteria. We divide the staining from weak to strong into three levels: 0 (negative), 1 (weakly positive), 2 (moderately positive), and 3 (strongly positive). The percentage scores: 0 (0% cells stained), 1 (1%-25% cells stained), 2 (26%-50% cells stained), and 3 (50%-100% cells stained). Multiply the scores of staining intensity and the percentage: the high expression group (0-4.5) and the low expression group (4.5-9).

### Western blot assay

Tissue specimens and cells were lysed in RIPA buffer (Beyotime, China) and the protein concentrations were measured by a BCA kit (Beyotime, China). Equal amounts of proteins were separated on 10% SDS-PAGE and transferred to polyvinylidene fluoride membranes. After washing and blocking with sealing fluid, all membranes were incubated with rabbit polyclonal anti-RIOK1 (1: 2,000 dilution, Abcam, Cambridge, UK), anti-Cyclin B1 (1:1,000 dilution, Cell Signaling Technology, #4135), anti-p-AKT Ser473 (1:1,000 dilution, Cell Signaling Technology, #4060), anti-AKT (1:1,000 dilution, Cell Signaling Technology, #4691), anti-MMP2 (1:1,000 dilution, Cell Signaling Technology, #40994), anti-N-cadherin (1:1,000 dilution, Cell Signaling Technology, #13116), anti-vimentin (1:1,000 dilution, Cell Signaling Technology, #5741), anti-Cleaved PARP (1:1,000 dilution, Cell Signaling Technology, #5625), anti-Cleaved Caspase-3 (1:1,000 dilution, Cell Signaling Technology, #9664), anti-stat3 (1:1,000 dilution, Cell Signaling Technology, #12640), anti-p-stat3 Tyr705 (1:1,000 dilution, Cell Signaling Technology, #9145), anti-twist (1:1,000 dilution, Cell Signaling Technology, #69366), anti-E-cadherin (1:1,000 dilution, Abcam, Cambridge, UK), Tubulin (1:1,000 dilution, Abcam, Cambridge, UK). And then the corresponding secondary antibodies were incubated. ECL kit was used for detection.

### MTT and Colony Formation Assays

The cell proliferation was evaluated by MTT and colony formation assays. The MTT assay was performed according to the manufacturer's instructions. For colony formation assay, cells were seeded with a density of 400 cells into 6 well plates. 400 cells were plated in 6-well plates and incubated at 37 °C in a 5% CO_2_ incubator. After 14 days of culture, cells were stained with crystal violet and colonies were counted.

### Cell Migration and Invasion Assays

For cell migration and invasion assays, 24-well Transwell chambers with an 8-mm pore size polycarbonate membrane were used (Corning Incorporated). Cells were seeded on the top of the membrane precoated with Matrigel (BD) (without Matrigel for the cell migration assay). After incubation for 24 hr, cells inside the upper chamber were removed with cotton swabs, whereas cells on the lower membrane surface were fixed and then stained with 0.5% Crystal violet solution. Five randomly selected fields were counted in each well.

### Cell Apoptosis

To determine cell apoptosis, H1299 and A549 cells were treated with cisplatin (16 μg/ml) for 24 hr, followed by incubation with APC and 7-AAD. The frequency of apoptotic cells was measured following the manufacturer's protocol for the Annexin V-APC/7-AAD kit (KeyGEN BioTECH, KGA1026).

### *In vivo* Tumor Growth Assay

All animal experiments were performed following the guidelines for ethical review of laboratory animal welfare (China National Standard GB/T 35892-2018) and were approved by the Animal Ethics Committee of Jinzhou Medical University. A total of 1×10^7^ A549 (scrambled shRNA or RIOK1-shRNA) cells in a volume of 200 μl PBS were injected subcutaneously in the right oxter of each 5-week-old BALB/c nude mouse (Vital River Laboratory Animal Technology Co. Ltd., Beijing, China). Tumor volumes were examined every 7 days after injection using the formula V= 1/2 length × width^2^. The mice were killed with anesthesia after a 35 days period of observation. Tumors were obtained and weighed.

### Statistical Analysis

The statistical analysis was performed using SPSS (17.0) software. The overall survival (OS) and recurrence-free survival (RFS) were estimated using the Kaplan-Meier method and analyzed by Log-Rank test. The RIOK1 expression correlated with NSCLC clinical character was tested by Pearson χ^2^. Statistical comparisons between only two groups were carried out using unpaired Student's t-test or the Mann-Whiney U test when a normal distribution could not be assumed. Data are presented as mean ± s.e.m. The results were defined significant if *p* < 0.05 (*p < 0.05, ** p < 0.01, *** p < 0.001).

## Results

### RIOK1 overexpressed in NSCLC tissues and correlated with poor prognosis of NSCLC patients

**Figure 1:**
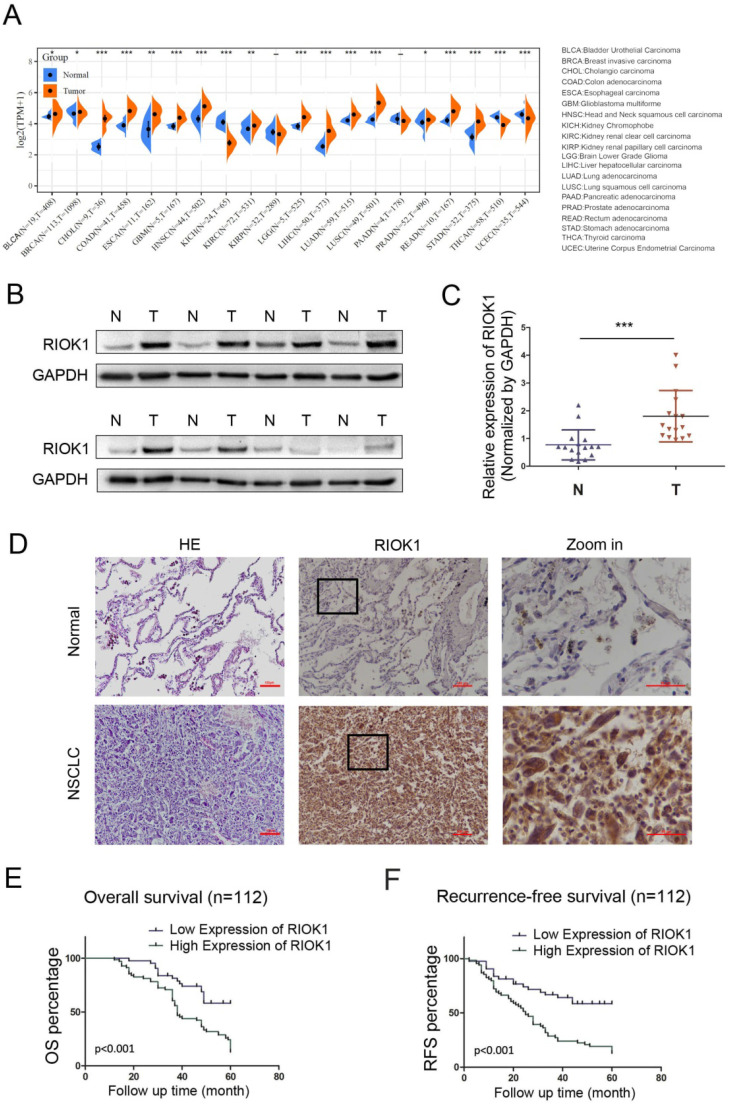
** RIOK1 expression in NSCLC and Kaplan-Meier survival analysis of NSCLC patients. (A)** The expression of RIOK1 in pan-cancer was examined in SangerBox. **(B)** The expression of RIOK1 in NSCLC and adjacent tissues was detected by Western blot. **(C)** Statistical analysis of gray value in Western blot; ***p <0.001. **(D)** Representative H&E and immunohistochemical staining of RIOK1 in NSCLC and adjacent normal tissues. **(E and F)** Kaplan-Meier curves of OS and RFS in patients with NSCLC.

The expression of RIOK1 in pan-cancer was examined in SangerBox and was generally elevated in cancers (Fig. 1A). Among them, we compared the mRNA expression of RIOK1 between lung cancer and normal tissues. The results indicated that the expression of RIOK1 was higher in cancer tissues than in normal tissues whether in LUAD or LUSC. To further confirm the expression level of RIOK1 in NSCLC tissues, using western bolt assays, we detected the expression level of RIOK1 in NSCLC tissues and matched adjacent normal tissues. Results showed that compared with matched adjacent normal tissues (N=16), RIOK1 has a significantly elevated expression in NSCLC tissues (N=16) (Fig. 1B and C and Fig. S1). Next, using immunohistochemical, we found that RIOK1 staining in NSCLC tissues mainly located at both nucleus and cytoplasm of the cancer cells, but negative RIOK1 expression was detected in normal lung tissues (Fig. 1D). Furthermore, compared to the relationship between clinicopathological factors and the expression of RIOK1 in 112 cases of NSCLC, As shown in Table 1, no difference was observed between RIOK1 expression level and patient age (*p*=0.951), gender (*p*=0.28), smoking history (*p*=0.821) or pathologic type (*p*=0.479). High RIOK1 expression level positively correlated with pathological grade (*p*<0.001), tumor size (*p*=0.049), lymph node metastasis (*p*=0.01) and survival status (*p*<0.001) of NSCLC (Table 1). In addition, the Kaplan-Meier survival curves were used to evaluate the relationship between the expression of RIOK1 and OS/RFS in NSCLC patients. These results exhibited that high expression of RIOK1 had significantly worse OS/RFS compared to the low RIOK1 group (Log-Rank test, *p*<0.05) (Fig. 1E and F). Moreover, we performed univariate and multivariate analyses to examine the relative risk of the prognostic parameters in NSCLC (Table 2). The Cox regression model showed that the RIOK1 expression level (hazard ratio 8.23; 95% confidence interval CI 0.239-0.764, P=0.004), pathologic stage (hazard ratio 6.191; 95% CI 0.308-0.869, *p*=0.013), and tumor size (hazard ratio 14.082; 95% CI 1.135-1.495, *p*<0.001) were independent prognostic factors for the poor overall survival of NSCLC patients.

### Knockdown of RIOK1 inhibits NSCLC cell proliferation

**Figure 2:**
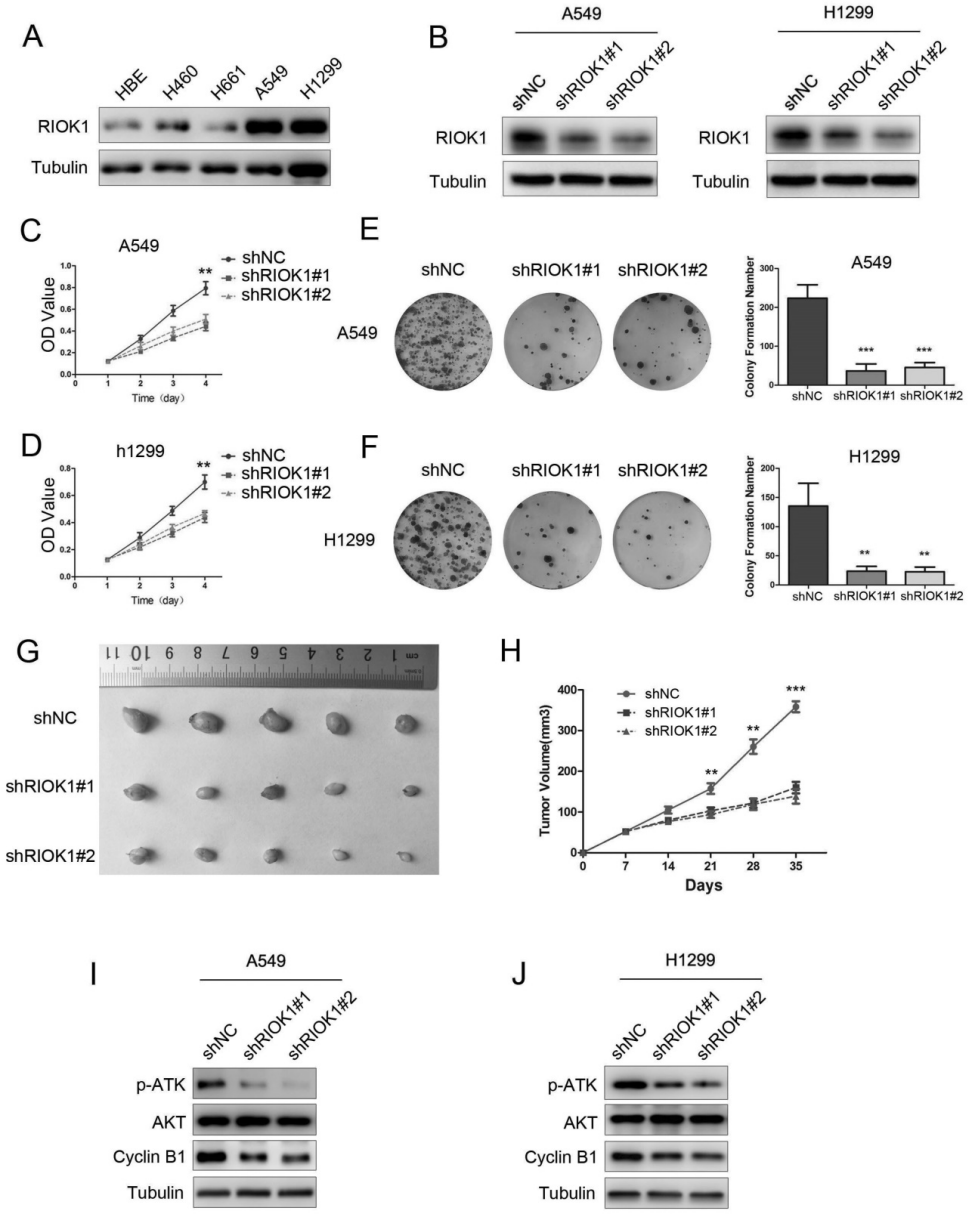
** Knockdown of RIOK1 inhibits NSCLC cell proliferation *in vitro* and *in vivo.* (A)** RIOK1 protein expression in HBE and four non-small cell lung cancer (NSCLC) cell lines. **(B)** Stable knockdown of RIOK1 in A549 and H1299 cell lines was detected by Western blot. **(C, D)** Cell proliferation was detected by MTT assays for up to 4 days. All data are presented as the mean ± s.e.m. from three independent experiments. **p < 0.01. **(E, F)** Cell colony forming assay showed that knockdown of RIOK1 declined the cell growth of (E) A549 and (F) H1299 cells. All data are presented as the mean ± s.e.m. from three independent experiments. **p < 0.01, ***p < 0.001. **(G)** Representative images of xenograft tumors excised from nude mice injected with shRNA-RIOK1 or shRNA NC A549 cells. **(H)** Comparison of tumor growth curves of the shRNA-RIOK1 groups and the NC group. **(I, J)** Western blot showing the expression of p-AKT, AKT and Cyclin B1 in (I) A549 and (J) H1299 cell lines. Tubulin served as protein loading control.

To investigate the functions of RIOK1 in NSCLC, we first used a western blotting assay to detect its expression in lung cancer cell lines. As observed in western blotting results, RIOK1 expression was high in A549 and H1299 cell lines, and low in the H460 and H661 cell lines (Fig. 2A and Fig. S2A). We selected A549 and H1299 cells lines for subsequent studies. The western blot assay exhibited that RIOK1 expression was significantly down-regulated in NSCLC cells transfected with RIOK1-specific shRNA (shRIOK1#1 and shRIOK1#2) compared with the shRNA negative control (Fig. 2B and Fig. S2B). The proliferation of tumor cells was evaluated by MTT assays, the growth curves showed that depletion RIOK1 significantly inhibited A549 and H1299 proliferation (Fig. 2C and D). The inhibitory effect of RIOK1 on NSCLC cells proliferation was further verified by colony formation assay. These results revealed that knockdown of RIOK1 significantly blocked the colony formation ability of NSCLC cells (Fig. 2E and F, **p<0.01, ***p<0.001). Moreover, we detected the proliferation ability of RIOK1 *in vivo*. RIOK1 knockdown and control A549 cells were subcutaneously injected into nude mice. We found that compared with the control group, silencing RIOK1 significantly decreased tumor growth *in vivo* (Fig. 2G). The mean weight of the tumors injected with RIOK1 knockdown cells was also significantly less than shNC groups (Fig. 2H, *p<0.05). Since RIOK1 inhibits cell growth, we monitored related proteins to identify affected pathways. We found that knockdown of RIOK1 caused down-regulated protein expression of p-AKT and Cyclin B1 (Fig. 2I and J). We also found that the expression of RIOK1 is positively related to the expression of p-AKT and Cyclin B1 in NSCLC tissues (Table S1 and Fig. S3).

### RIOK1 depletion suppressed cell migration and invasion in NSCLC cells

**Figure 3:**
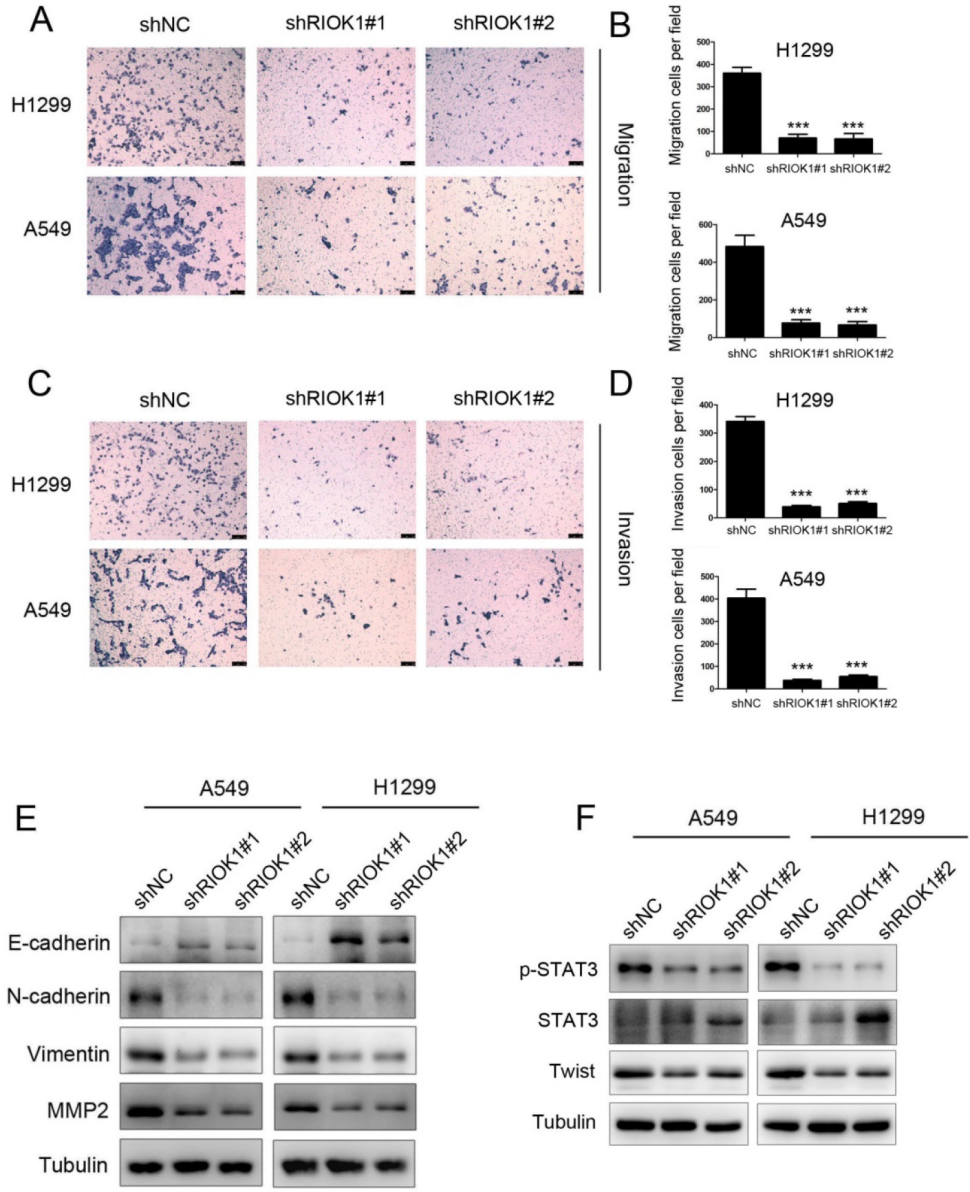
** RIOK1 depletion suppressed cell migration and invasion in NSCLC cells. (A-D)** Knockdown of RIOK1 inhibited H1299 and A549 cell migration (A-B) and invasion (C-D). All data are presented as the mean ± s.e.m. from three independent experiments. ***p<0.001. **(E)** Western blot showing the expression of E-cadherin, N-cadherin, vimentin and MMP2 in A549 and H1299 cells. Tubulin served as a protein loading control. **(F)** Western blot showing the expression of p-STAT3, STAT3, and Twist in A549 and H1299 cells. Tubulin served as a protein loading control.

As RIOK1 knockdown inhibited cell proliferation, whether cell migration and invasion were involved was examined by transwell assays subsequently. The results showed that the migration ability of silencing RIOK1 H1299 and A549 cells was significantly lower than that of the control group (Fig. 3A and B). This result was consistent with that of the invasive transwell experiment (Fig. 3C and D). Collectively, these findings indicate that knockdown of RIOK1 expression inhibited the migration and invasion of NSCLC cancer cells. Since the epithelial-mesenchymal transition broadly regulates invasion and metastasis by acquiring cellular traits associated with high-grade malignancy, we speculated that the acquisition of migration and invasion potential by RIOK1 might depend on induction of the EMT program. To address the issue, we examined whether RIOK1 regulates an EMT program in NSCLC. We found that knockdown of RIOK1 caused up-regulated E-cadherin and downregulated N-cadherin, vimentin, as well as MMP2 (Fig. 3E). During the process of tumor metastasis, a set of EMT-TFs orchestrate the EMT program and related migratory processes. These recent studies and the above observation led us to speculate that RIOK1 might contribute to the induction of EMT via the up-regulation of EMT-TFs. To test this possibility, we examined the expression of a set of EMT-TFs. Among them, STAT3 activation, as well as Twist-1 expression, was markedly decreased in the RIOK1-shRNA cells relative to control-shRNA cells; however, STAT3 expression was not affected in the knockdown of RIOK1. We also found that the expression of RIOK1 is positively related to the expression of MMP2 and Twist in NSCLC tissues (Table S1 and Fig. S3).

### RIOK1 confers cisplatin resistance to NSCLC cells

**Figure 4:**
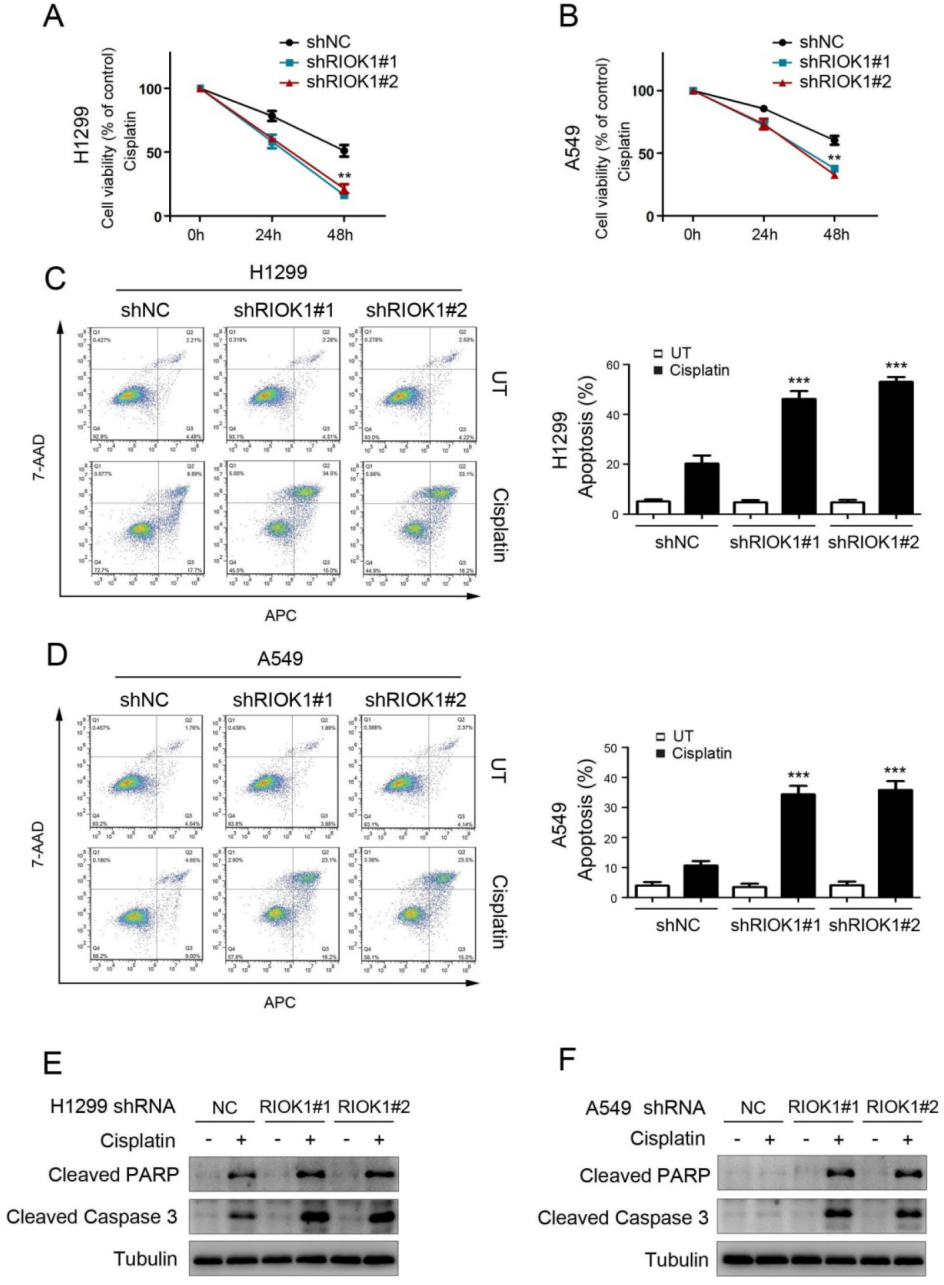
** RIOK1 confers cisplatin resistance to NSCLC cells. (A and B)** MTT assay showed that RIOK1 depletion significantly decreased H1299 (A) and A549 (B) cells viability following 24 and 48 hours of cisplatin treatment (16 µg/ml). All data are presented as the mean ± s.e.m. from three independent experiments. **p<0.01. **(C-D)** Cell apoptosis assays showed that RIOK1 depletion significantly up-regulated apoptosis rate in H1299 (C) and A549 (D) cells. All data are presented as the mean ± s.e.m. from three independent experiments. ** *p*<0.01. **(E and F)** Western blot showing the expression of cleaved PARP and cleaved Caspase-3 in H1299 (E) and A549 (F) cells. Tubulin served as a protein loading control.

To investigate the effect of RIOK1 on the chemical resistance of NSCLC cells, we treated cancer cells with cisplatin and performed cell viability analysis to check the survival rate. Compared with control cells, silencing RIOK1 inhibited the resistance of H1299 cells and A549 cells to cisplatin, resulting in decreased cell viability (Fig. 4A and B). We further confirmed the role of RIOK1 in the chemical resistance of NSCLC cells through apoptosis experiments. As shown in Figures 4C and D, compared with control cells, silencing RIOK1 significantly increased apoptosis rate in H1299 and A549 cells following 24 hours of cisplatin treatment. Moreover, we found that RIOK1 depletion caused up-regulated protein expression of cleaved PARP and cleaved Caspase-3 (Fig. 4E and F). Taken together, RIOK1 supports cisplatin resistance in NSCLC cells.

## Discussion

RIOK1 is upregulated in 8% of primary cancer cells suggesting it as a potential biomarker and anti-cancer drug objective 18, 23. The RIOK1 has been reported to be over-expressed in gastric adenocarcinoma, cecal adenocarcinoma, colorectal adenoma, and colorectal adenocarcinoma, suggesting that RIOK1 engages in a functional relationship with constitutively active RAS to regulate the tumor cell malignancy 14. Over-expression of RIOK1 was further found in HR-negative breast cancer and correlated with advanced stage and poorer breast cancer patient prognosis. Overexpression of RIOK1 was shown to promote breast cancer cells proliferation and invasion by regulating the PI3K/AKT and MAPK/ERK signaling pathways in HR-negative breast cancer cells 17. RIOK1 has been reported to be highly expressed in NSCLC 23, however, its clinical significance and biological function are not clear.

In this study, using western blot and immunohistochemistry, we showed that RIOK1 was highly expressed in NSCLC tissues. To investigate the relationship between the expression of RIOK1 and clinicopathological characteristics in NSCLC patients, we found that the expression of RIOK1 was correlated with pathological grade, tumor size, lymph node metastasis, and survival status of NSCLC (*p< 0.05, Table 1). Furthermore, the Kaplan-Meier survival curves were used to evaluate the relationship between the high and low expression of RIOK1 and OS/DFS in NSCLC patients (*p*<0.05). These results exhibited that the high expression of RIOK1 had significantly worse OS/RFS compared to the low RIOK1 group. These results were consistent with the previous study on the upregulation of RIOK1, which was associated with the progression and low survival rate of breast cancer. In summary, these findings revealed a novel function of RIOK1 in non-small cell lung cancer progression.

Next, we want to explore whether RIOK1 affects the biological function of NSCLC cells. Knockdown of RIOK1 could inhibit cell growth rate, colony formation, migration, and invasion in NSCLC cells and tumorigenesis *in vivo*. Accordingly, the protein level of Cyclin B1 and phosphorylation of AKT were down-regulated after the knockdown of RIOK1. That is consistent with previous studies that the knockdown of RIOK1 significantly rewards the protein level of Cyclin B1 and phospho-AKT in breast cancer cells 17. Basement membrane degradation and epithelial-mesenchymal transition (EMT) are considered to be important steps of metastasis 24-27. In this study, we found that knockdown of RIOK1 reduced the protein levels of MMP2, the mesenchymal marker N-cadherin and vimentin. Meanwhile, we found that RIOK1 knockdown increased the expression of epithelial marker E-cadherin. During the process of tumor metastasis, a set of EMT-TFs orchestrate the EMT program and related migratory processes 28-31. These recent studies and the above observation led us to speculate that RIOK1 might contribute to the induction of EMT via the up-regulation of EMT-TFs. Among them, STAT3 activation, as well as Twist-1 expression, was markedly decreased in the RIOK1-shRNA cells relative to control-shRNA cells. These results suggest that RIOK1 silencing reduces the migration and invasion of NSCLC cells by inhibiting EMT. More importantly, we found that RIOK1 plays an important role in the drug resistance of NLCSC cells. Cell viability and apoptosis assays demonstrated that RIOK1 maintained NSCLC cell survival and reduced apoptosis rate when cells were treated with cisplatin. Western blot analysis demonstrated that RIOK1 depletion caused upregulated protein expression of cleaved PARP and Caspase-3 in H1299 and A549 cells. Here, we showed that RIOK1 can cause phenotypic changes such as lung cancer cell proliferation, migration, and invasion, and is accompanied by changes in these phenotypic important executive proteins. However, the direct effectors downstream of RIOK1 still needs to be further revealed.

In conclusion, our study for the first time clarified RIOK1 positively correlated with the malignant progression of lung cancer, and the high expression of RIOK1 indicates a poor prognosis. It is a further step for the research of RIOK1 in lung cancer. These findings revealed the roles and possible molecular mechanisms of RIOK1 in NLCSC cell proliferation, migration, invasion, and drug resistance and suggested that RIOK1 might become a promising diagnostic and therapeutic target for this disease.

## Supplementary Material

Supplementary figures and tables.

## Figures and Tables

**Table 1:** Distribution of RIOK1 status in NSCLC according to clinicopathological characteristics

Characteristics	Low or none, no cases	High, no cases	*p* value
Age (mean ± SD)	61.79±1.426	61.90±1.042	0.951
**Gender**			
Female	20	25	0.28
Male	23	44	
**Smoking history**			
Yes	19	32	0.821
No	24	37	
**Pathologic stage**			
I-II	33	31	0.002*
III-IV	10	38	
**Pathologic type**			
AC	36	54	0.479
SCC	7	15	
Tumor size (mean ± SD)	2.791±0.247	3.459±0.215	0.049*
**Nodal invasion**			
Yes	13	38	0.01*
No	30	31	
**Survival stage**			
Dead	17	58	<0.001*
Live	26	11	

**Table 2:** Univariate and multivariate analyses of molecular and clinical factors with prognosis of NSCLC patients

Variables	Univariate analysis			Multivariate analysis		
	HR	95%CI	*p* value	HR	95%CI	*p* value
Age	0.434	0.968-1.016	0.51	1.344	0.959-1.011	0.246
Gender	5.976	0.331-0.885	0.015	0.221	0.484-1.560	0.638
Smoking history	1.644	0.472-1.170	0.2	0.013	0.595-1.792	0.909
Pathologic stage	23.547	0.2-0.505	<0.001*	6.191	0.308-0.869	0.013*
Pathologic type	0.22	0.486-1.556	0.639	1.568	0.348-1.262	0.21
Tumor size	21.984	1.169-1.462	<0.001*	14.082	1.135-1.495	<0.001*
Nodal invasion	10.402	0.296-0.743	0.001*	0.912	0.474-1.293	0.34
RIOK1	15.937	0.192-0.57	<0.001*	8.23	0.239-0.764	0.004*

Note: *P<0.05 was considered statistically significant;Abbreviations: CI, confidence interval; HR, hazard ratio.

## Abbreviations

AKT: Protein Kinase B
EMT: Epithelial Mesenchymal Transition
ERK: Extracellular Regulated protein Kinases
IHC: Immunohistochemical
LUAD: Lung Adenocarcinoma
LUSC: Lung Squamous Cell Carcinoma
MMP: Matrix Metallopeptidase
NSCLC: Non-Small-Cell Lung Cancer
OS/RFS: Overall survival/Disease-free survival
RIOK1: The right open reading frame kinase1
STAT3: Signal Transducer and Activator of Transcription 3
