# Supplementary material for: RIOK1 is associated with non-small cell lung cancer clinical characters and contributes to cancer progression
Source: J Cancer. 2022 Jan 31;13(4):1289–98. doi: 10.7150/jca.64668 (PMC8899362; doi:10.7150/jca.64668)
Supplement: Supplementary file 1 — Supplementary figures and tables. [file jcav13p1289s1.pdf]

Figure S1

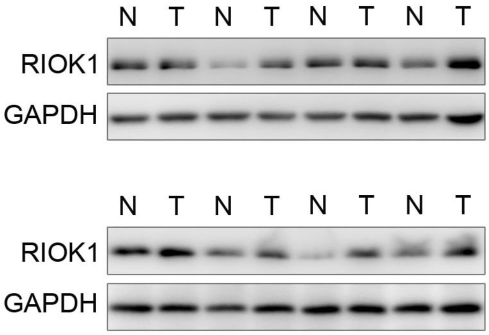

Figure S2

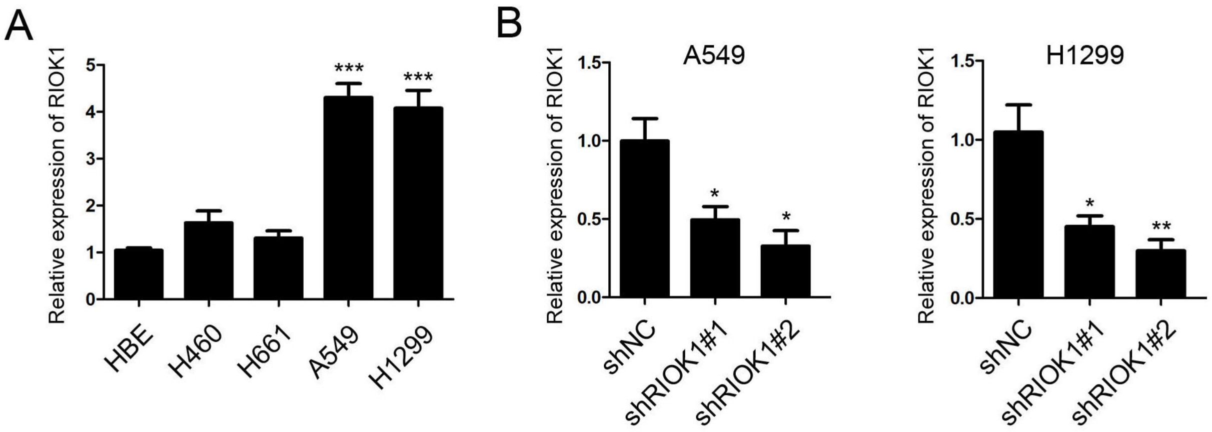

A
Relative expression of RIOK1
5
4
3
2
1
0
***
***
HBE
H460
H661
A549
H1299
B
A549
Relative expression of RIOK1
1.5
1.0
0.5
0.0
*
*
shNC
shRIOK1#1
shRIOK1#2
H1299
Relative expression of RIOK1
1.5
1.0
0.5
0.0
*
**
shNC
shRIOK1#1
shRIOK1#2

Figure S3

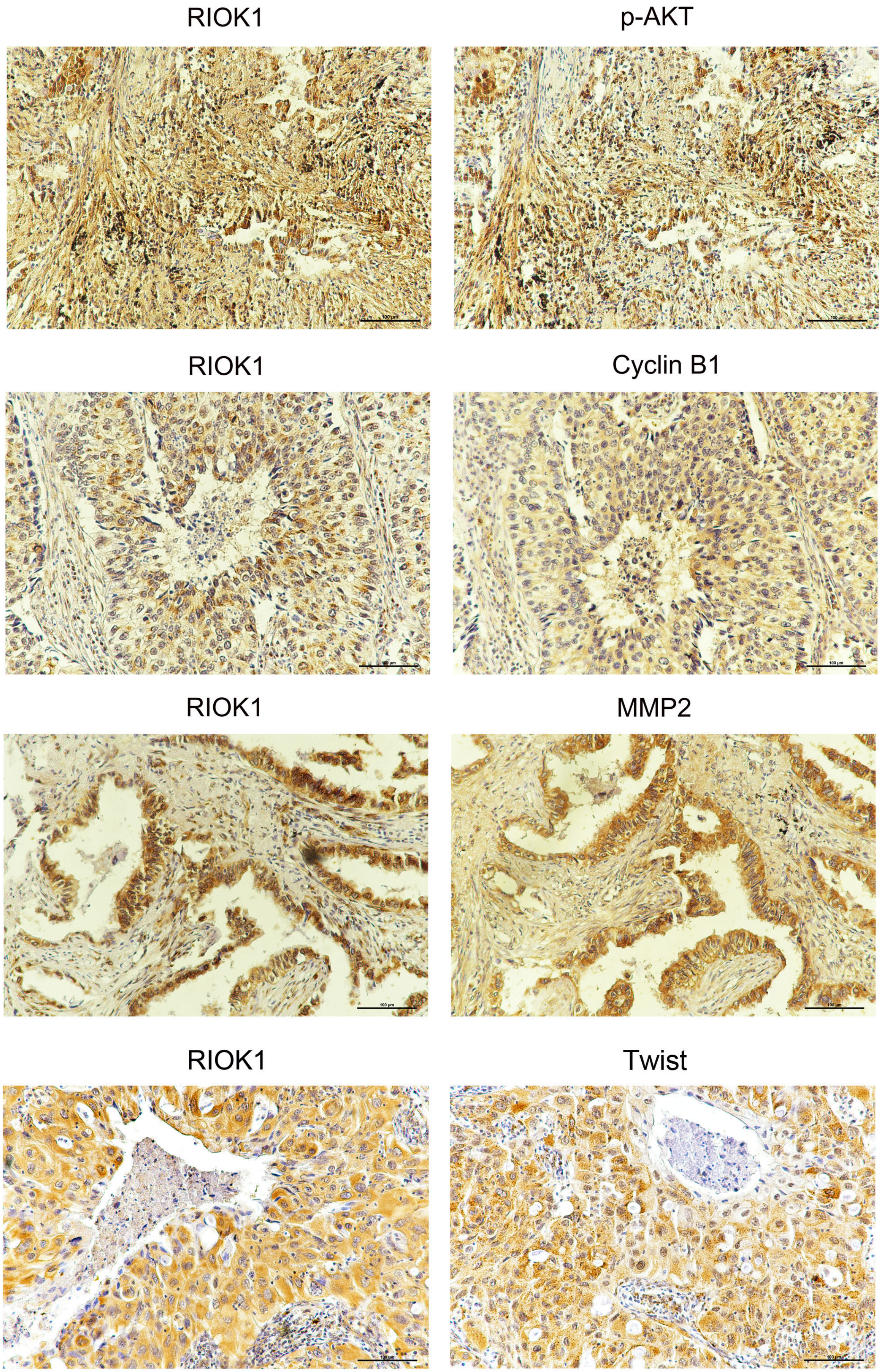

Table 2 Correlation between riok1 and p-AKT, MMP2, CyclinB1 and Twist protein expression in patients with NSCLC

		Expression of RIOK1			
		**Low (%)**	**High (%)**	**$\chi^{2\text{-value}}$**	***p*-value**
**Expression of p-AKT**	**Low (%)**	34 (77.27)	10 (22.73)	46.32	<0.001
	**High (%)**	9 (13.24)	59 (86.76)		
**Expression of MMP2**	**Low (%)**	30 (50)	30 (50)	7.36	0.007
	**High (%)**	13 (25)	39 (75)		
**Expression of CyclinB1**	**Low (%)**	27 (45.76)	32 (54.24)	2.86	0.91
	**High (%)**	16 (30.19)	37 (69.81)		
**Expression of Twist**	**Low (%)**	35 (74.47)	12 (25.53)	44.56	<0.001
	**High (%)**	8 (12.31)	57 (87.69)		

Supplementary Figure legends

Figure S1. RIOK1 expression in NSCLC and adjacent tissues.

The expression of RIOK1 in NSCLC and adjacent tissues was detected by Western blot.

Figure S2. RIOK1 expression in NSCLC cell lines.

(A) Quantification of RIOK1 protein expression in HBE and four non-small cell lung cancer (NSCLC) cell lines. Results from three independent experiments, ***P < 0.001.

(B) Quantification of RIOK1 protein expression in A549 and H1299 cell lines was detected by Western blot. Results from three independent experiments, *P < 0.05, **P < 0.01.

Figure S3. Immunohistochemical analyses of RIOK1, p-AKT, MMP2, Cyclin B1, and Twist in NSCLC.

The protein expression between RIOK1 and p-AKT, MMP2, Cyclin B1, and Twist in the same NSCLC sample.
